# Ubiquitin E3 ligases in the plant Arg/N-degron pathway

**DOI:** 10.1042/BCJ20240132

**Published:** 2024-12-13

**Authors:** Keely E. A. Oldham, Peter D. Mabbitt

**Affiliations:** Scion, Titokorangi Drive, Private Bag 3020, Rotorua 3046, New Zealand

**Keywords:** plant signal transduction, protein turnover, structural biology, ubiquitin signalling

## Abstract

Regulation of protein longevity via the ubiquitin (Ub) — proteasome pathway is fundamental to eukaryotic biology. Ubiquitin E3 ligases (E3s) interact with substrate proteins and provide specificity to the pathway. A small subset of E3s bind to specific exposed N-termini (N-degrons) and promote the ubiquitination of the bound protein. Collectively these E3s, and other N-degron binding proteins, are known as N-recognins. There is considerable functional divergence between fungi, animal, and plant N-recognins. In plants, at least three proteins (PRT1, PRT6, and BIG) participate in the Arg/N-degron pathway. PRT1 has demonstrated E3 ligase activity, whereas PRT6 and BIG are candidate E3s. The Arg/N-degron pathway plays a central role in plant development, germination, and submersion tolerance. The pathway has been manipulated both to improve crop performance and for conditional protein degradation. A more detailed structural and biochemical understanding of the Arg/N-recognins and their substrates is required to fully realise the biotechnological potential of the pathway. This perspective focuses on the structural and molecular details of substrate recognition and ubiquitination in the plant Arg/N-degron pathway. While PRT1 appears to be plant specific, the PRT6 and BIG proteins are similar to UBR1 and UBR4, respectively. Analysis of the cryo-EM structures of Saccharomyces UBR1 suggests that the mode of ubiquitin conjugating enzyme (E2) and substrate recruitment is conserved in PRT6, but regulation of the two N-recognins may be significantly different. The structurally characterised domains from human UBR4 are also likely to be conserved in BIG, however, there are sizeable gaps in our understanding of both proteins.

## Introduction

A large proportion of plant proteins are dedicated to ubiquitin (Ub) signalling [1]. The ubiquitin E3 ligases (E3s) recruit both substrates and ubiquitin conjugating enzymes (E2s) and in doing so provide the majority of the specificity to signalling pathways. The largest families of E3s (Really Interesting New Gene (RING), and UFD2-homology domain (U-box)) act as binding sites for E2s and often activate the E2 for Ub transfer [2]. In contrast, several families of E3 including Homologous to the E6-AP Carboxyl Terminus (HECT), RING-Between-RING (RBR), and RING-Cys-Relay (RCR) have catalytic cysteines and act in multi-step mechanisms to receive Ub from the E2 and subsequently catalyse Ub transfer to substrates [3–9]. There are likely more mechanistically distinct E3s across biology [4,5,10,11].

For many E3s the catalytic machinery and substrate recruitment domains can be predicted from sequence analysis alone. In contrast, identification of bona fide E3 substrates is challenging as the interactions between E3s and substrates are typically weak or transient [12]. The substrates for the majority of the ∼1400 predicted E3s in Arabidopsis are unknown [1,13,14]. The number of predicted E3s in economically important crop and forestry species is variable and only a small sample of E3s from non-model species have been investigated. Detailed structural and molecular understandings of substrate recognition and ubiquitination are becoming increasingly important as investigators seek to exploit ubiquitination to improve agronomic traits [15–18]. There are a range of biotechnological approaches to manipulating ubiquitin signalling in plants. For example, proteins can be engineered so that they are conditional substrates of endogenous E3s, this allows for controlled knockdown of the target protein [18,19]. An interesting approach is to fuse temperature dependent degradation tags to cytotoxic proteins allowing for accumulation of the protein under permissive temperatures. In principle this approach could be used for production of high-value toxic proteins [19]. Controlled protein depletion can also be achieved by introducing an inducible orthogonal-E3 and fusing the orthogonal-E3's recognition sequence to target proteins [20].

In plants, as in other eukaryotes, recognition of substrate proteins with destabilising amino-terminal sequences (N-degrons) is mediated by specialised E3 ligases and other proteins which are collectively known as N-recognins [21–25]. The Arg/N-degron pathway was first described in the context of recognition of N-terminal arginine but is now used to describe all non-acetylated N-degrons [21]. The substrate preferences of plant Arg/N-recognins have diverged from their fungi and animal counterparts. A plant-specific N-recognin known as PROTEOLYSIS 1 (PRT1) recognises aromatic (Phe, Trp, and Tyr) N-termini [26,27]. The E3 ligase activity of PRT1 with authentic substrates has been demonstrated *in vitro* [28–31]. N-degrons with positively charged N-termini (Arg, Lys, His) are bound by a UBR1-like protein known as PROTEOLYSIS 6 (PRT6) [22]. The degradation of both PRT1 and PRT6 substrates is enhanced by an N-recognin known as BIG [24]. Despite the fundamental importance of the Arg/N-degron pathway in plants there has been slow progress in structurally and biophysically characterising N-recognins. Much of our current understanding of the plant Arg/N-degron pathway is informed by studies of yeast and human proteins. In this perspective we highlight recent advancements in the structural biology of N-recognins and discuss new directions for probing the plant Arg/N-degron pathway.

## Generation and recognition of N-degrons

**Figure 1.**
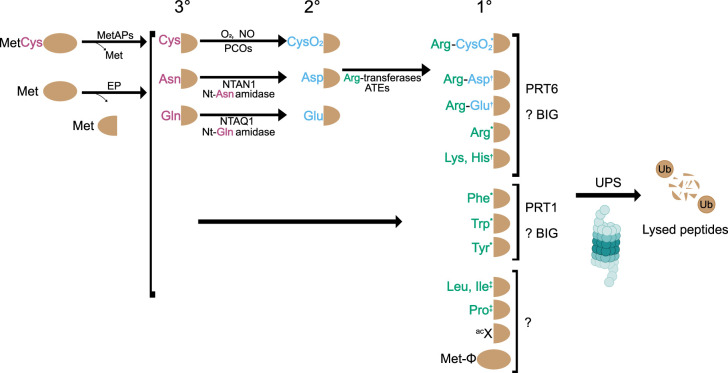
Overview of Arg/N-degron pathways present in plants. Tertiary (3°), secondary (2°) and primary (1°) destabilising residues are coloured magenta, blue and green, respectively. Alternate N-degron pathways are shown in black text. Methionine aminopeptidases (MetAPs) or endopeptidases (EP) cleave proteins resulting in new N-termini. Primary destabilising residues are directly recognised by PRT1 or PRT6. A third N-recognin, BIG, enhances the activity of both PRT1 and PRT6. The secondary destabilising residue CysO2 (cysteine sulfinic acid) is the product of Cys oxidation with molecular oxygen (O2) by PLANT CYSTEINE OXIDASEs (PCOs). Cysteine can also be oxidised non-enzymatically via nitric oxide (NO) and reactive oxygen species (ROS). Arg-tRNA transferases (Arg-transferases, ATEs) attach an Arg to secondary destabilising residues. The tertiary destabilising residues Asn and Gln are deamidated to Asp and Glu by specific amidases (NTAN1 and NTAQ1). Evidence for N-degron presence in plants is annotated with a * (physiological substrates), † (model substrates, responsible N-recognin is known or inferred based on preferences of homologous mammalian N-recognin) or (‡ model substrates, responsible N-recognin is unknown). Abbreviations: UPS, ubiquitin proteasome system; acX, acetylated N-degron. Figure modified from [24,137].

Plant genomes encode hundreds of proteases and the majority of these do not have known physiological substrates [32]. While it is likely that non-processive endopeptidases produce N-degrons in plants there are few examples of this in the literature [33,34]. The majority of known plant N-degrons are produced as the result of removal of the N-terminal Met by methionine aminopeptidases (MetAPs) (Figure 1) [35–37]. N-degrons are categorised as either primary, secondary or tertiary destabilising residues, reflecting the need for additional modification for recognition by N-recognins [21]. In the Arg/N-degron pathway, primary residues include type I positively charged residues (Arg, His, Lys) and type II hydrophobic residues (Phe, Tyr, Trp, Leu and Ile) (Figure 1). Amino-terminal Asn and Gln are classified as tertiary destabilising residues, which undergo deamination catalysed by Nt-Asn amidase (NTAN1) and Nt-Gln amidase (NTAQ1), respectively [38,39]. Additionally, Cys is a tertiary destabilising residue, undergoing oxidation by PLANT CYSTEINE OXIDASE (PCO) to produce cysteine sulfinic acid (CysO_2_) [40]. Secondary destabilising residues (Asp, CysO_2_, and Glu) undergo arginylation catalysed by Arg-tRNA transferase (in *Arabidopsis* ATE1/ATE2) yielding a primary Arg N-degron [35] (Figure 1). There is evidence suggesting the Leu/Ile N-degrons are destabilising in plants [22,39], but the responsible N-recognins remain elusive. Similarly N-recognins for acetylated/N-degron [41], Gly/N-degron [42], Pro/N-degron [43] and formyl-Met/N-degron [44] pathways have not been identified in plants.

**Figure 2.**
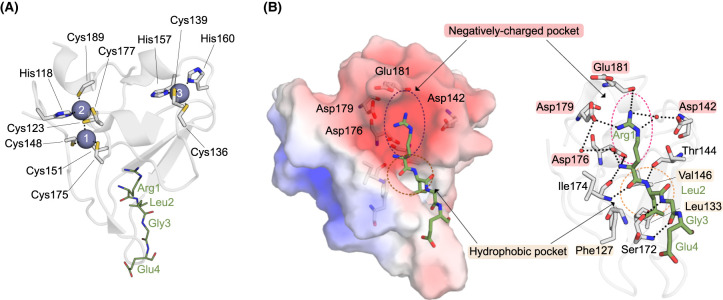
UBR-box from Saccharomyces UBR1 (PDB 3NIN). (**A**) Cartoon representation of the UBR-box contains two zinc fingers (grey sticks) coordinating three zinc ions via a series of Cys and His residues. (**B**) Surface charge map and a cartoon representation of the UBR-box, residues forming the negatively charged pocket and hydrophobic pockets are shown as sticks. Key pocket residues are annotated. N-degron peptide (Arg1-Leu2-Gly3-Glu4) is shown as green sticks. Zinc ions are shown as purple spheres. Zinc and peptide coordinating residues are shown as sticks. Surface charge map was created using the Adaptive Poisson-Boltzmann Solver plug-in in PyMOL [138]. Hydrogen bonds are indicated with dashes.

A 70-residue domain known as a Ubiquitin-Recognin (UBR) box binds to Arg/N-degrons [45–48]. The archetypical UBR-box domains from Saccharomyces UBR1 and mammalian UBR1 and UBR2 bind to type I N-degrons [45–48] (Figure 2). The UBR-box adopts a distinctive-fold comprised of two zinc fingers that co-ordinate three zinc ions (Figure 2A) [45,46]. The α-amino group of the substrate hydrogen bonds with an Asp side chain (Asp176 in Saccharomyces UBR1) that is present in all UBR-box domains [45,46]. UBR-box domains co-crystalised with Arg/N-degron peptides reveal that acidic residues (Asp142, Asp176, and Asp179 in Saccharomyces UBR1) form a negatively charged pocket that accommodates the sidechain of Arg (Figure 2B) [45,46]. The side chain of the residue at the second position in the N-degron is directed towards a shallow hydrophobic pocket. This results in a modest preference for hydrophobic residues at the second position [45,49]. Saccharomyces UBR1 and mammalian UBR1 and UBR2 recognise type II N-degrons via a ClpS-like domain known as the N-domain [50]. As detailed in later sections, plants use an alternate recognition domain to bind type II N-degrons [31]. Once an N-degron substrate is bound, an exposed lysine needs to come into close proximity with an E2-Ub conjugate (or E3-Ub conjugate) in order for Ub transfer to occur [21]. As a consequence, proteins with a flexible or disordered N-terminus are more likely to be substrates of N-recognins [21,51].

### Ubiquitin conjugating (E2) enzymes in the Arg/N-degron pathway: lessons from animal and yeast models

The largest families of E3s (RING and U-box) recruit and allosterically activate E2s for Ub transfer [2]. All of the currently characterised plant Arg/N-recognins either have RING domains or are predicted to have RING-like domains [22–24,52]. Ubiquitin is typically transferred from E2s to lysine residues within substrate proteins. The amino-terminus and seven lysine residues of Ub are also targets for ubiquitination. The distinct ubiquitin chain linkages produced by the ∼40 E2s have a range of biological roles which have been reviewed extensively elsewhere [53–56]. In the plant Arg/N-degron pathway, the only biochemically validated interaction between ab E2 and an E3 is between PRT1 and UBC8 [29,31]. The interactions between non-plant N-recognins and E2s provide a framework for investigating the potential E2 recruitment domains of PRT6 and BIG.

Animal and yeast N-recognin proteins appear to have high specificity for a small subset of E2s [52,57–62]. The sole Arg/N-recognin in Saccharomyces (UBR1) interacts with UBC2 (also known as RAD6) [51,57]. Apart from a repeat of acidic residues at the C-terminus, Saccharomyces UBC2 is highly similar to Arabidopsis UBC1 and UBC2, and human UBE2A and UBE2B (Supplementary Figure S1). The almost identical sequences of the UBE2A and UBE2B paralogues make them difficult to distinguish in mass spectrometry experiments [59]. For convenience we will refer to the paralogues collectively as UBC2.

**Figure 3.**
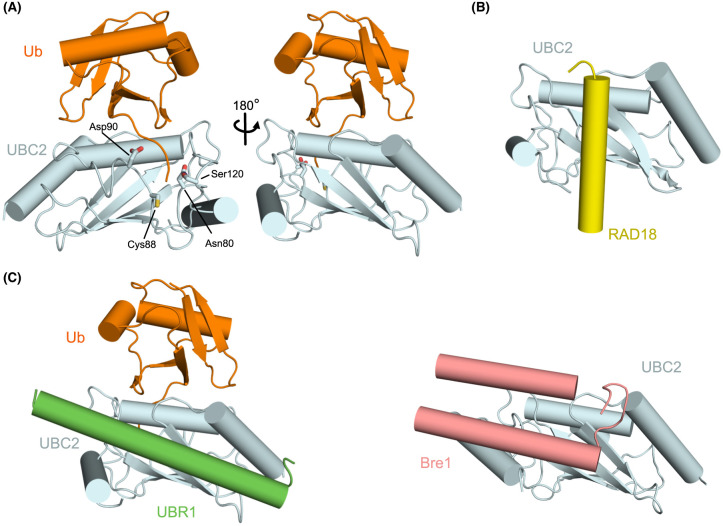
The ubiquitin conjugating enzyme UBC2 participates in the yeast and animal Arg/N-degron pathways. (**A**) The folded-back state of the UBC2-Ub conjugate observed in a cryo-EM structure of UBR1 (PDB 7MEX). In an active E2 the catalytic cysteine (Cys88) is linked by a thioester bond to the C-terminus of Ub, residues within the E2 (Asn80, Asp90, and Ser120) enhance the rate of Ub transfer to substrates. (**B**) An alpha-helix of Rad18 binds to the backside of UBC2 (yellow: PDB 2YBF) and reduces the Ub chain building activity of the E2. (**C**) Both UBR1 (green; PDB 7MEX) and Bre1 (pink; PDB 7UV8) bind to the backside of UBC2.

E2-Ub conjugates are conformationally flexible and the orientation of the Ub-thioester relative to key residues in the E2 active site is important for Ub transfer [63–65]. In the cryo-EM structures of the UBC2-Ub conjugate bound to Saccharomyces UBR1, the UBC2-Ub conjugate is in the folded-back state (Figure 3A) [51]. The folded-back conformation is generally considered to be necessary for efficient transfer of Ub to lysine [64,66]. Mutagenesis and *in vitro* activity assays support the conclusion that the folded-back conformation of UBC2-Ub is required for lysine aminolysis [52,67].

UBC2 forms Ub chains in the absence of an E3. The E3-independent activity of UBC2 is enhanced by non-covalent binding of Ub to the ‘backside’ of UBC2 [68,69]. Non-covalent interactions between E2s and Ub are relatively weak (reported *K*_D_ of between 0.3 and 1 mM) and the precise mechanism by which these interactions enhance activity, in the absence of a RING domain, is not clear [69,70]. When RING domains are present, non-covalent Ub binding can have a more than additive (positive cooperative) effect on Ub discharge [70,71]. Alpha-helices from E3 ligases compete with Ub for backside binding and in some instances reduce the chain building activity of UBC2 (Figure 3B) [68]. For at least two E3s (UBR1 and Bre1) the backside interaction with UBC2 provides the majority of the binding interface (Figure 3C) [51,68,72–74]. Notably for the UBR1-UBC2 interaction, the affinity of the backside binding helix (named the UBC2-binding region; U2BR) is reported to be two thousand-fold greater (*K*_D_ of ∼140 nM) than the affinity of UBC2 for Ub [51]. The U2BR has a large number of positively charged residues which potentially interact with the negatively charged C-terminus of Saccharomyces UBC2 [75], and it is possible that electrostatic interactions contribute to the unusually tight binding of the yeast proteins. As discussed in later sections PRT1 and BIG may also interact with UBC2 via a backside binding helix.

### Ubiquitin ligases (E3) in the Arg/N-degron pathway

#### PRT1

**Figure 4.**
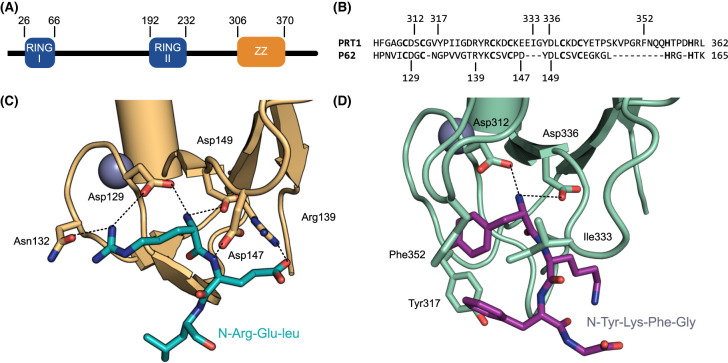
The ZZ-type zinc finger domain of PRT1 is similar to that of the mammalian p62/sequestosome 1. (**A**) Domain architecture of Arabidopsis PRT1. (**B**) Amino acid sequence alignment of the ZZ-domains of PRT1 (UniProt accession number Q8LBL5) and P62 (UniProt accession number Q13501). Putative zinc coordinating residues are in bold. Residues in p62 and PRT1 that are involved in substrate recognition are indicated. (**C**) Close-up of the p62 ZZ domain (wheat) 217 with a type I N-degron bound (Arg-Glu-Leu, cyan sticks) (PDB 6MIU). (**D**) AlphaFold3 model of the PRT1 ZZ domain (green) with a type II N-degron bound (Tyr-Lys-Phe-Gly, purple sticks) [31,139]. Hydrogen bonds are indicated with dashes.

PRT1 was identified in a genetic screen for mutants which stabilised a reporter with an N-terminal Phe [26,27]. Subsequently it was found that Arabidopsis PRT1 can recognise and degrade proteins with aromatic (Phe, Trp, and Tyr) N-termini in a Saccharomyces *ubr1* knockout strain; this suggests that PRT1 is a single subunit E3 ligase that does not require a plant-specific binding partner [23]. The PRT1 protein has two RING domains and a single ZZ-type zinc finger domain (Figure 4A) [31]. *In vitro* ubiquitination assays revealed that PRT1 interacts with UBC8 and can polyubiquitinate model N-degron substrates [76]. AlphaFold2 models and *in vitro* activity data suggest that the RING domains of PRT1 form an intramolecular dimer [31]. As was proposed in 2018, the ZZ domain of PRT1 acts in a similar way to the ZZ domain of the mammalian autophagy adaptor p62/sequestosome 1 (Figure 4B) [31,77]. Both ZZ domains are structurally similar to the prototypical UBR-box domain [31,77]. The p62 ZZ domain preferentially binds to N-terminal Arg and also binds aromatic N-termini, but with at least 10-fold lower affinity (Figure 4C) [77–79]. In contrast, the PRT1 ZZ domain has a clear preference for bulky hydrophobic substrates [31]. In both p62 and PRT1 a pair of Asp side chains (Asp312 and Asp336 in PRT1) form hydrogen bonds with the alpha-amino group of the substrate (Figure 4C,D) [77]. Three residues in PRT1 (Ile333, Tyr317, Phe352) provide specificity for aromatic N-termini (Figure 4D). Individual mutation of these residues to alanine impairs substrate binding and ubiquitination [31].

Proteins involved in pathogen resistance are up-regulated in *prt1* knockout plants and this contributes to altered resistance to bacterial and fungal pathogens [76,80]. It is possible that up-regulation of pathogen resistance proteins is an indirect result of perturbation of the N-degron pathway, but this is still under debate. Understanding the role of PRT1 in pathogen resistance will require identification of its specific substrates. At present the only characterised substrate of PRT1 is the E3 ligase BIG BROTHER (BB). The metalloprotease DA1 cleaves BB resulting in an N-terminal Tyr which is targeted by the N-degron pathway [33]. The BB protein is stabilised, when transiently expressed with an engineered Tyr N-terminus, in *prt1* protoplasts [33]. Identifying other physiological substrates of PRT1 remains a major challenge. Recent crystal structures of PRT1 suggest that a hydrophobic network contributes to a preference for hydrophobic residues at the third position of the degron [31]. The degree to which the third position of the N-degron contributes to PRT1 binding *in vivo* is unknown.

#### PRT6

**Figure 5.**
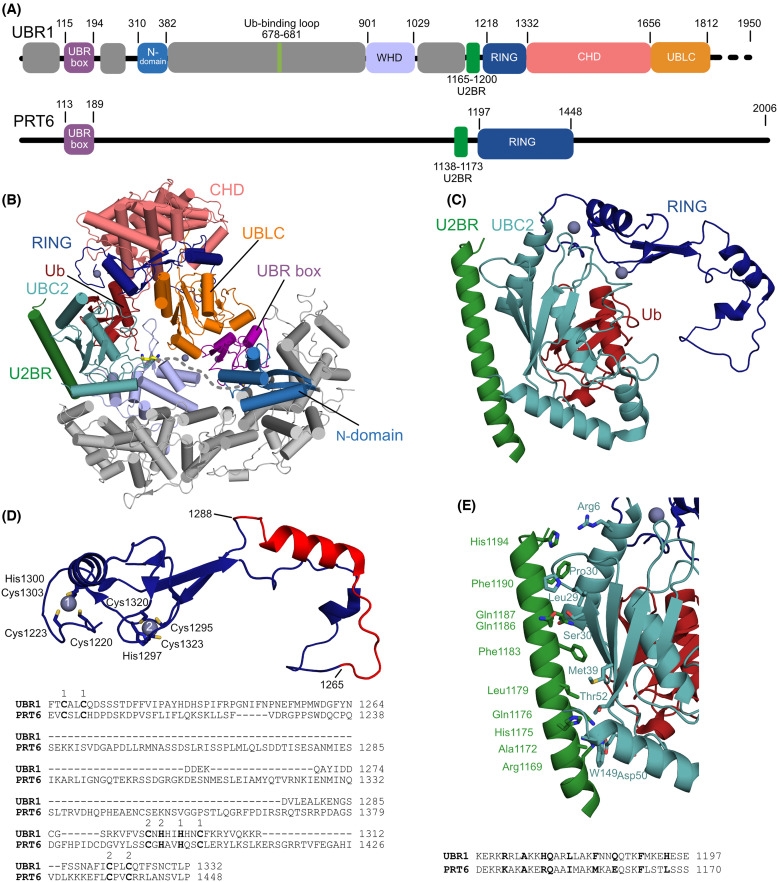
Comparison of Saccharomyces UBR1 and Arabidopsis PRT6. (**A**) Domain architecture of UBR1 [51] and the putative domain architecture of PRT6. Abbreviations: WHD, winged helix domain; U2BR, UBC2-binding region; CHD, cap helix domain; UBR/Leu/Cys, UBLC) domain. Helical scaffold regions are coloured grey. (**B**) Cartoon representation of the cryo-EM structure of the Saccharomyces UBR1 UBC2-Ub-degron complex (PDB 7MEX). The substrate is bound by the UBR-box and cross-linked to the UBC2-Ub conjugate. The residues linking the substrate lysine (Lys17, yellow sticks) and the N-terminus were not resolved and are indicated with grey dashes. (**C**) Close-up of the interface between the U2BR, RING, and UBC2-Ub conjugate. Residues linking the U2BR and RING domain were not resolved in the structure. (**D**) The RING domains of PRT6 and UBR1 are similar. Cartoon representation of the RING domain from Saccharomyces UBR1. Zinc coordinating residues are shown as sticks. An amino acid sequence alignment of the RING domains suggests that the zinc coordination residues, indicated in bold, are conserved. The region linking the two zinc fingers (UBR1 residues 1265 to 1288, coloured red) is much larger in PRT6. (**E**) Interface between U2BR and UBC2. Residues at the U2BR-UBC2 interface are shown as sticks. An amino acid sequence alignment of the U2BR regions from UBR1 and PRT6. Residues at the interface are in bold.

Whereas PRT1 is restricted to the green lineage [76], PRT6 appears to be an orthologue of UBR1 and has a number of well characterised substrates. Despite having low sequence identity (∼15% pairwise identity) Arabidopsis PRT6 has a similar domain architecture to Saccharomyces UBR1 (Figure 5A, Supplementary Figure S2A). The shared domain architecture is readily apparent in AlphaFold models of PRT6 [81] (Supplementary Figure S2B). Key regions of the PRT6 protein have large insertions and deletions, relative to UBR1. These regions include the RING domain and the ClpS-like domain (N-domain) (Figure 5A, Supplementary Figure S2A) [22,50,51]. The N-domain of UBR1 has sequence and structural similarity to bacterial and chloroplast ClpS proteins [25,51,82,83]. The binding pocket of these proteins has a contiguous Asn-Asp-Asp motif that is disrupted in PRT6 (Supplementary Figure S3). Consistent with disruption of the N-domain, PRT6 degrades model substrates with Arg, but not Leu or Phe, N-termini [22].

The cryo-EM structures of Saccharomyces UBR1 in complex with UBC2, Ub and an Arg N-degron peptide highlight some key similarities and differences between the PRT6 and UBR1 signalling complexes (Figure 5B) [51]. The RING domain of PRT6 has large loop insertions relative to UBR1. The positions of these loop insertions suggest that they would be unlikely to impact UBC2 binding or activation (Figure 5C,D). The short UBC2-binding region (U2BR) precedes the RING domain of UBR1 and interacts with the backside of UBC2 [32]. The U2BR is present in PRT6, and the periodicity of the helix suggests that it would form a near identical interaction with UBC2 (Figure 5E).

Several elements identified in the cryo-EM structure of UBR1 are absent in PRT6. The Ub-binding loop (His678-His681) of UBR1 comes into close proximity with the acceptor Ub during Ub-chain elongation. N-degron ubiquitination is reduced when all four residues of the Ub-binding loop are mutated to alanine [51]. Sequence alignments suggest that the entire Ub-binding loop is absent from PRT6 (Supplementary Figure S2). Similarly, the winged helix domain (WHD) and cap helix domain (CHD) of UBR1 come into close proximity with the donor Ub. Mutation of WHD: Lys965 to Ala and CHD: Glu1436 and Gln1437 to Ala has a modest negative effect on ubiquitin chain elongation [51]. Regions with sequence similarity to the WHD and CHD are present in PRT6 but the specific Ub interacting residues are not conserved (Supplementary Figure S2). The UBR/Leu/Cys (UBLC) domain contains a zinc finger (Cys1703, Cys1706, His1722, Cys1727) which is likely conserved in PRT6 [51,84]. It has been proposed that the UBLC acts as a regulatory domain that blocks a binding site on UBR1. This blockage is alleviated when both the UBR-box and N-domain are occupied by N-termini [84]. The UBLC occupies a central position in the UBR1 complex consistent with it having a regulatory role (Figure 5A,B). A putative zinc coordination site provides a three-way junction between the UBR box (His161), WHD (Asp952) and the UBLC domain (His1763, Asp1775) of UBR1, but these residues are not conserved in PRT6 (Supplementary Figure S2). Altogether, these observations suggest that the regulation of PRT6 may differ significantly from Saccharomyces UBR1.

The Arg/N-degron pathway is required for diverse developmental processes in Arabidopsis. These include responses to oxygen levels, optimum seed germination, control of leaf and shoot morphology, and leaf senescence [35,85,86]. The best characterised PTR6 substrates are the group VII Ethylene Response Factor (ERFVII) transcription factors. These transcription factors have a Cys residue (Cys2) immediately preceded by the N-terminal Met. Proteolytic removal of the amino-terminal Met by MetAPs results in an N-terminal Cys. Oxidation of the Cys and subsequent arginylation by ATE1 produces PRT6 substrates [35]. The levels of both O_2_ and NO determine whether the N-terminal Cys is oxidised [36]. Under normoxic conditions PCO enzymes catalyse the conversion of N-terminal cysteine to cysteine-sulfinic acid (CysO_2_) [40]. The apparent affinities of the four most active Arabidopsis PCOs (PCO1, 2, 3, and 4) for O_2_ suggests that they may act redundantly as oxygen sensors *in planta* [87]. The recognition of an N-terminal Cys peptide, rather than a small molecule, sets PCOs apart from other cysteine dioxygenases [88–90]. It is yet to be determined if PCOs discriminate between different N-terminal sequence motifs *in vivo*. In principle the substrate specificity of PCOs could be engineered in order to improve plant responses to hypoxia (e.g. improved submergence tolerance) [91]. However, given the modest affinity (µM Michaelis-Menten constant (*K_M_*)) of Arabidopsis PCOs for N-terminal peptides this would not be a trivial task. There is evidence that N-terminal cysteine-sulfinic acid (CysO_2_) is further oxidised to cysteine-sulfonic acid (CysO_3_) *in vivo* [92]. It is unclear if the second oxidation occurs before or after arginylation or if it affects PRT6 binding [92]. A recent study investigated the interaction between the PRT6 UBR-box and Arg-Asp-Gly substrates [93]. Aspartic acid acts as a proxy for negatively charged CysO_2_ at the second position of the degron. Similar to orthologous UBR-box domains, the PRT6 UBR-box can accommodate a range of amino acids, including Asp, at the second position [45,49,93].

Early results suggest that manipulation of the Met-Cys branch of the Arg/N-degron pathway may be a viable route for crop improvement. Knockdown of barley (*Hordeum vulgare*) *PRT6* increases the plant's tolerance to waterlogging, salinity and drought stress [17,94]. Overexpression of rice (*Oryza sativa*) ERFVII transcription factors (SUB1A-1, ERF66, or ERF67) in submergence-sensitive cultivars results in enhanced submergence tolerance [95]. The ERF66 and ERF67 transcription factors act downstream of SUB1A-1 and are regulated by the Arg/N-degron pathway. Despite having an N-terminal Met-Cys, SUB1A-1, is not regulated by the Arg/N-degron pathway. The C-terminal region of SUB1A-1 appears to prevent recognition of the N-terminus [95]. A number of proteins have been identified that, like SUB1A-1, have N-terminal destabilising residues but are not Arg/N-degron substrates *in vivo* [96,97]. A detailed molecular explanation for this substrate discrimination would assist in identifying and engineering N-degron substrates.

#### BIG

Arabidopsis with mutations in the *BIG* gene show a range of morphological and developmental defects including; altered root architecture, reduced organ size, and delayed flowering [98–103]. Morphological and severe developmental defects are also observed in tomato and rice *big* mutants, suggesting that BIG may play similar roles in these species [104,105]. The size of the *BIG* gene (a 15 234 base-pair open reading frame, encoding a putative 5077 amino acid protein) has been a significant hurdle to detailed biochemical investigations of BIG [98,106]. Most descriptions of BIG come from forward genetic screens where the gene is interrupted and presumably the protein is not expressed [99–103,106–108]. The *big* allele *doc1-1* (dark overexpression of CAB), first characterised by the Chory group, is unusual as a point mutation results in a Cys to Tyr substitution in the UBR-box of BIG [98,109]. The mutated Cys is central to the UBR-box and likely co-ordinates two zinc ions [45]. The transcript levels of *BIG* in *doc1-1* plants appear to be similar to those in wild-type plants but protein expression data is currently unavailable [98,106]. If BIG protein is produced by *doc1-1* plants, then it is likely defective in Arg/N-degron binding but it may retain other functional domains. It is possible that truncated BIG proteins produced by mutagenesis or alternative splicing have distinct biological functions, but to our knowledge this has not been investigated.

It has been evident for over 20 years that BIG has sequence similarity with E3 ligases, but direct biochemical evidence that BIG has E3 ligase activity is lacking [98,110]. In 2024 Zhang et al. [24] used N-degron substrates with Arg N-termini fused to a biotin ligase to identify proteins which preferentially interact with N-degrons. Both PRT6 and BIG were found to interact with Arg N-degrons, however, it should be noted that this could be an indirect interaction where BIG is part of a larger N-degron recognition complex. Knockout of *big* did not significantly stabilise model N-degron substrates, however, *big* had an additive effect on substrate stability in both a *prt1* and a *prt6* background. Furthermore, mutation of *big* in a *prt6* background enhanced the stability of two transcription factors (HRE2 and VRN2) which are regulated by the Met-Cys branch of the Arg/N-degron pathway [24]*.* Given the similarity between mammalian UBR4 and BIG UBR-box domains it is likely that the UBR-box of BIG binds to both Arg and Phe N-termini (Supplementary Figure S4) [50,111].

**Figure 6.**
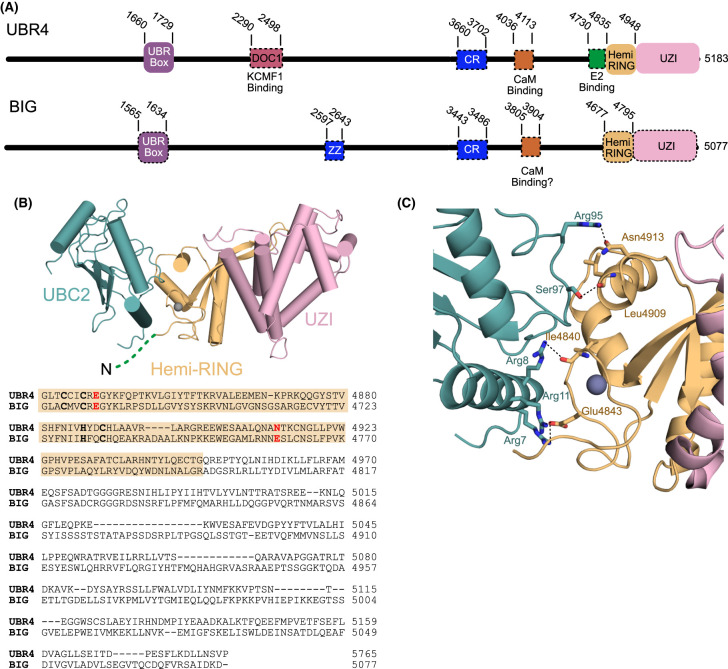
Comparison of UBR4 and BIG. (**A**) Domain architecture of human UBR4 and Arabidopsis BIG. Abbreviations DOC (destruction of cyclin B), KCMF1 (potassium channel modulatory factor 1), ZZ (ZZ-type zinc finger), CR (cysteine rich), CaM (calmodulin), UZI (UBR zinc-finger interacting). Domains with dashed lines have not been structurally characterised. (**B**) Structure of the UBR4 Hemi-RING and UZI in complex with UBC2 (PDB 8BTL). The amino-terminal residues of UBR4 (4730–4832) were unresolved in the structure. An amino acid sequence alignment of UBR4 (UniProt accession number Q5T4S7) and BIG (UniProt accession number Q9SRU2) highlighting residues that co-ordinate zinc (bold) or interact with UBC2 (bold, red). (**C**) Close-up of the interface between UBC2 and the Hemi-RING. Residues at the interface are shown as sticks. Hydrogen bonds are indicated with dashes.

The BIG protein has ∼20% sequence identity with mammalian UBR4 and Drosophila Poe (Purity of essence, also known as Calossin and Pushover) [58,98,112,113]. Several regions of the proteins have higher sequence identity. These regions include the UBR-box [45,110,111], a cysteine rich (CR) domain [98], as well as the recently identified Hemi-RING and UZI (UBR zinc-finger interacting) domain [52] (Figure 6A, Supplementary Figure S4). Mammalian UBR4 has a well-defined calmodulin (CaM) binding region [58,114,115], which appears to be present in BIG. A direct association between BIG and CaM has not been established. A region of BIG has similar sequence to the PRT1 and p62 ZZ-domains [98] (Figure 6A, Supplementary Figure S4). It remains to be determined if the BIG ZZ-domain interacts with N-degrons.

Mammalian UBR4 has E3 ligase activity and a C-terminal region of UBR4 recruits UBC2 with high specificity [52]. Mammalian UBR4 and Drosophila Poe also interact with an E3 ligase known as KCMF1 (potassium channel modulatory factor 1) via a DOC1-like (destruction of cyclin B) domain [58,59,62,116,117] (Figure 6A). Direct interactions between UBR4 and the proteasome have also been observed, further supporting the idea that UBR4 is part of a large signalling complex [118,119]. In mammals a complex of UBR4, KCMF1, and calmodulin, named the silencing factor of the integrated stress response (SIFI) recognises and degrades unimported mitochondrial proteins that remain in the cytosol and are prone to deleterious aggregation [58]. The SIFI complex recognises alpha-helical regions within mitochondrial matrix targeting sequences and the UBR-box is not required for recognition of these substrates [58]. The SIFI also degrades stress response components; this is important for the return of cellular homeostasis after specific stressors have been removed [58]. The finding that the UBR-box is not essential for all of UBR4's cellular functions should provide new avenues for exploring the biochemical function of BIG. Notably, engineered UBR4 without a UBR-box can be transcribed and translated into a functional signalling complex [58]. This suggests that disruption of the UBR-box in *doc1-1,* or similar mutants, could also result in a partially functional signalling complex [98,106].

In mammalian cells UBR4 interacts with UBC2 [52,58,59]. A minimal region required for UBC2 recruitment and Ub transfer activity was identified at the C-terminus of UBR4 [52]. This region contains a single zinc-finger (Hemi-RING) and an adjacent UBR zinc-finger interacting (UZI) region. The Hemi-RING is structurally analogous to a RING domain, and the mode of UBC2 binding is similar to that observed in other RING-E2 complexes [52] (Figure 6B). An elongated loop of the Hemi-RING packs against the UZI region and this appears to be required for folding of the E3 module. Immediately N-terminal of the Hemi-RING is a region that is predicted to be alpha-helical but has not been structurally characterised. This N-terminal region increases UBR4's affinity for UBC2 and may be binding to the backside of UBC2. Surprisingly, the isolated Hemi-RING did not significantly increase the rate of Ub discharge from UBC2 [52]. This suggests that either allosteric activation of UBC2 is not necessary in the context of UBR4 or that the region used in the *in vitro* reconstitution experiments lacked a component required for allosteric activation. The zinc-coordinating residues of the Hemi-RING are present in Arabidopsis BIG. Two Hemi-RING residues were shown to be important for UBC2 binding to UBR4 [52]. The first residue, Glu4843 hydrogen bonds with two Arg side chains in UBE2A (Arg7 and Arg11). These residues are identical in BIG and Arabidopsis UBC2 (Figure 6C). The second residue, Asn4913 hydrogen bonds with UBC2 Arg95. In Arabidopsis the equivalent residues are BIG Glu4760 and UBC2 Gln95 (Figure 6C). A pair of conserved UBC2 residues, Arg8 and Ser97, hydrogen bond with the backbone of the Hemi-RING (Figure 6C). Mutation of either of these residues to Ala impairs UBC2 binding [52].

The structural details of KCMF1 binding to UBR4 have not been elucidated and there are no KCMF1 orthologues annotated in the Arabidopsis genome. It is possible that BIG interacts directly with other E3 ligases and proteomic experiments are likely to uncover these interactions [24]. If, as sequence similarity suggests, BIG is an E3 ligase that recognises and ubiquitinates substrate proteins that harbour N-degrons we might expect to observe changes at the proteome level in *big* mutants [24,52,58]. Numerous studies have reported phenotypic and transcriptional changes in *big* mutants. However, as discussed below, these phenotypes have not been directly linked to the E3 ligase activity of BIG. *BIG* was first identified in a mutant that accumulated transcripts of chlorophyll a-b binding protein in the dark [98,99]. Subsequently, several groups have identified roles for *BIG* in polar auxin transport and responses to phytohormones [98,100,103,107,120]. Notably, the trafficking of PIN-FORMED auxin transporter 2 (PIN2) between the plasma membrane and endosomes was less inhibited in *big* mutants co-treated with auxin and Brefeldin-A (a toxin that interferes with endosome trafficking) relative to the wild type [120]. Recent studies have revealed that Brefeldin-A treatment leads to a number of artefacts that complicate interpretation of these results [121,122]. A re-evaluation of PIN2 abundance, ubiquitination, and localisation in *big* mutants may help resolve if BIG directly interacts with PIN2 [62,63].

While there is evidence that mammalian SIFI binds to and ubiquitinates mitochondrial proteins, the available evidence for similar direct regulation of mitochondrial proteins by BIG is inconclusive [58,108]. Inhibition of mitochondrial complex III activity by antimycin A serves as a model for mitochondrial stress. The abundance of Alternative Oxidase 1a (AOX1a) increases under stress conditions and AOX1a is often used as a marker of mitochondria to nucleus (retrograde) signalling in plants [123]. After antimycin A treatment, both AOX1a transcript and protein levels are elevated in *big* mutants relative to the wild type [108]. There appears to be reciprocal regulation of the pathways which lead to the induction of auxin and AOX1a. Due to this reciprocal regulation, accumulation of AOX1a protein in *big* mutants may be an indirect result of auxin signalling defects rather than a direct result of BIG's E3 ligase activity [108].

Structural biology and targeted gene-editing are likely to delineate which domains of BIG are required for substrate recruitment and ubiquitination. It will be a major step forward to associate any of the substrate recruitment domains of BIG with specific phenotypes. There is much still to be discovered about the role of BIG in enhancing the Arg/N-degron pathway [24]. At present it is unknown if BIG recruits additional E3 ligases, primes substrates with Ub or extends Ub chains. A first step towards resolving this would be disrupting the Hemi-RING UBC2 interaction in Arabidopsis.

#### UBR7

**Figure 7.**
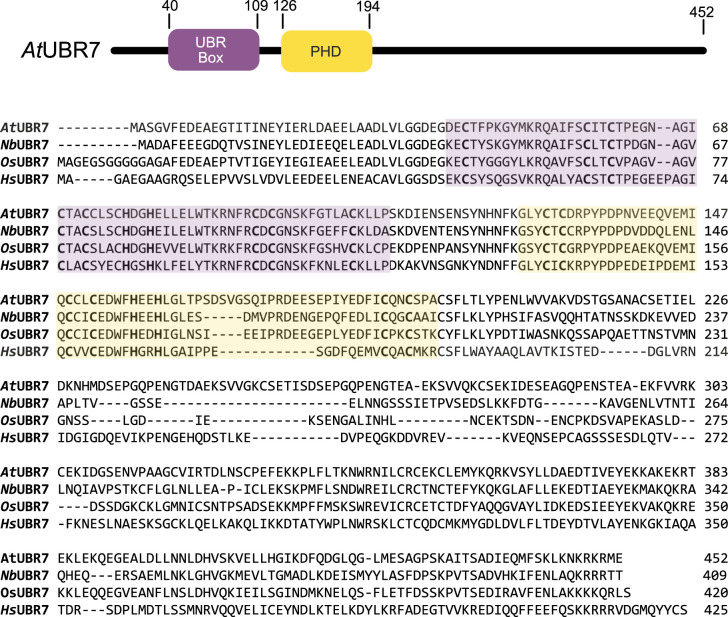
Domain architecture of Arabidopsis UBR7. An amino acid sequence alignment of *Arabidopsis thaliana* (*At*UBR7), *N. benthamiana* (*Nb*UBR7), *O. sativa* (*Os*UBR7) and *Homo sapiens* (*Hs*UBR7) indicates that the zinc coordinating residues (bold) of the UBR-box (purple) and atypical PHD domain (yellow) are conserved. UniProt accession numbers are AtUBR7, Q9T0A1; *Os*UBR7, Q0DBT4; and *Hs*UBR7, Q8N806. The *Nb*UBR7 sequence is reported in [128].

Three mammalian UBR-box proteins (UBR3, UBR6, and UBR7) do not bind to N-degrons under conditions where UBR1 binds [110,124–126]. Of these three, only UBR7 has apparent orthologues in plants. The UBR7 proteins from *O. sativa* (*Os*UBR7), *Nicotiana benthamiana* (*Nb*UBR7), and Arabidopsis (*At*UBR7) have similar domain organisations to mammalian UBR7 [110,127–129] (Figure 7). UBR7 has both an atypical plant homeodomain (PHD) zinc finger and a UBR-box domain. The PHD domain is structurally similar to a RING domain, however, most PHD domains bind to the N-terminus of histones [130,131]. In the context of full-length signalling proteins, the PHD domain can serve additional roles. For example, the two PHD domains of PHF7 enhance E2 binding to the RING domain of the protein [132]. There is biochemical evidence that the PHD domain of human UBR7 recruits both a histone substrate and an E2 (UBCH6) [126,133]. Empirical structural models of the UBR7-E2 complex would help to clarify the role of the PHD domain in E2 recruitment and activation. Pull-down experiments indicate that the UBR-box is not essential for histone binding, but it does contribute to the strength of the UBR7-histone interaction [126,134].

Three studies have reached somewhat disparate conclusions about the functions of UBR7 in plants. In tall rice cultivars knockout of *OsUBR7* leads to semi-dwarfism (a positive agronomic trait in rice and other cereals) and manipulation of *OsUBR7* expression may be useful in rice breeding [127]. *Os*UBR7 is constitutively expressed and localised to the nucleus. Mass spectrometry of proteins co-immunoprecipitated with *Os*UBR7 revealed a number of potential substrates including histone proteins [127]. In activity assays *Os*UBR7 ubiquitinated histone 2B, consistent with it having a similar function to human UBR7. An earlier study, in *N. benthamiana*, uncovered a potential role for UBR7 in pathogen resistance*.* A nucleotide-binding leucine-rich repeat immune receptor known as N is involved in resistance to tobacco mosaic virus (TMV). The N-protein fused to a biotin ligase was used to label interaction partners for subsequent identification by mass spectrometry. One of the N protein's putative interaction partners was *Nb*UBR7. Knock-down of *NbUBR7* stabilised the N protein and increased the plant's resistance to TMV [128]. The authors did not investigate the E3 ligase activity of *Nb*UBR7 or the regions of the proteins required for the interaction, however, *in vitro* pull-down assays did indicate a direct protein-protein interaction between N and *Nb*UBR7 [128].

A recent study suggests that *At*UBR7 is localised to the nucleus, where it acts as an N-recognin for an autophagy-related protein (ATG8a) [129]. In Arabidopsis ATG8a is proteolytically cleaved to reveal an Arg N-terminus (Arg13) which is not destabilised by PRT6 or BIG. Both co-immunoprecipitation and *in vitro* pull-down experiments suggest that *At*UBR7 binds to the nascent N-terminus of ATG8a, presumably via the UBR-box. The PHD domain was required for Arg13-ATG8a ubiquitination *in vitro* [129]. Overall, the emerging picture is that UBR7 is a predominantly nuclear-localised E3 ligase that recruits an E2 via an atypical PHD domain. Substrate recruitment is achieved, at least in part, via the UBR-box. It is likely that biophysical approaches will reveal the details of the unusual mechanism of UBR7.

## Conclusions

Beyond the UBR-box and ClpS-like domains it appears that a range of atypical zinc fingers are involved in Arg/N-degron recognition and ubiquitination. These atypical zinc fingers include the ZZ domain of PRT1, Hemi-RING of BIG, and the atypical PHD domain of UBR7. It would not be surprising if other zinc fingers that do not fall into established classes act as N-recognins in plants. This underscores the importance of biophysically validating interactions and not excluding candidates based on domain annotations.

The search for authentic substrates of N-recognins has been less fruitful in plants than other lineages, despite the growing sophistication of mass spectrometry workflows for enrichment of N-termini [34]. For example, the removal of PRT6 does not result in large global changes in the Arabidopsis proteome [135,136]. It is possible that the proteases which produce Arg/N-degrons are only active in specific tissues or under specific environmental conditions [137]. High-throughput chemical screens may be useful for identifying new phenotypes and Arg/N-degron substrates.

The post-translational regulation of PRT1, PRT6 and BIG activity has not been investigated. The presence of an autoinhibitory domain in UBR1 and a calmodulin-binding site in UBR4 suggest that N-recognin activity is subject to regulation [58,84,114,115]. Binding and activation of the E2-Ub conjugate is one potentially rate-limiting step in substrate degradation. Non-RING regions of both UBR1 and UBR4 have high affinity for the backside of UBC2 [42,43], and it is likely that PRT6 and BIG also form extensive interactions with the backside of UBC2. A peptide or small molecule that blocks the backside of UBC2 would be expected to inhibit both N-recognins and to stabilise ERFVII transcription factors.

## Abbreviations

AOX1a: Alternative Oxidase 1a
CHD: cap helix domain
CR: cysteine rich
MetAP: methionine aminopeptidase
PHD: plant homeodomain
TMV: tobacco mosaic virus
WHD: winged helix domain
