# Supplementary material for: Ubiquitin E3 ligases in the plant Arg/N-degron pathway
Source: Biochem J. 2024 Dec 13;481(24):1949–65. doi: 10.1042/BCJ20240132 (PMC11668491; doi:10.1042/BCJ20240132)
Supplement: Supplementary Material 1 [file BCJ-481-1949-s1.pdf]

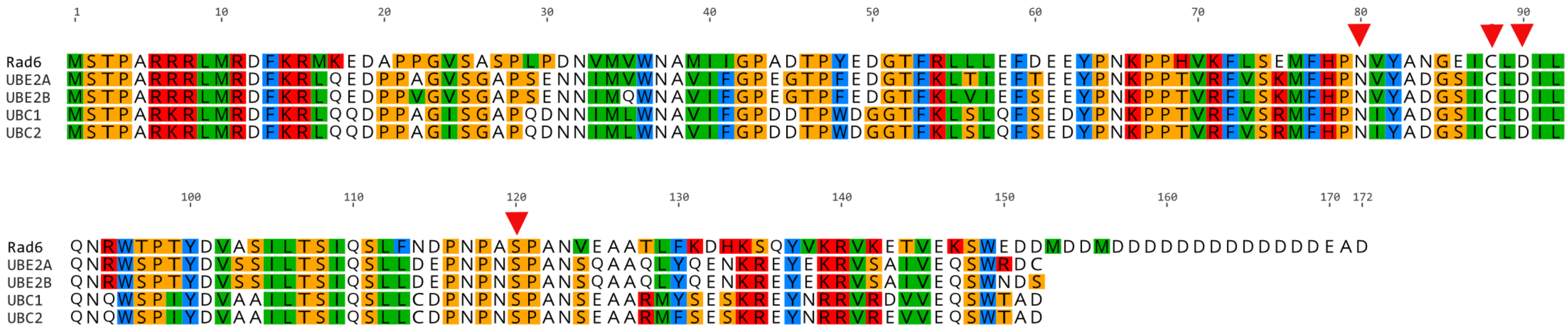

Supplementary Figure 1. Amino acid sequence alignment of Saccharomyces RAD6, with Human UBE2A and UBE2B, and Arabidopsis UBC1 and UBC2. The catalytic cysteine (Cys88) and residues that enhance Ub discharge (Asn80, Asp90 and Ser120) are indicated with red arrows. Residues are coloured green (Ile, Leu, Met, Val), orange (Gly, Pro, Ser, Thr), blue (Phe, Trp, Tyr) and red (Arg, His, Lys). The sequences and UniProt accession numbers are *Saccharomyces cerevisiae* RAD6 (P06104), *Homo sapiens* UBE2A (P49459), *H. sapiens* UBE2B (P63146), *Arabidopsis thaliana* UBC1 (P25865) and *A. thaliana* UBC2 (P42745).

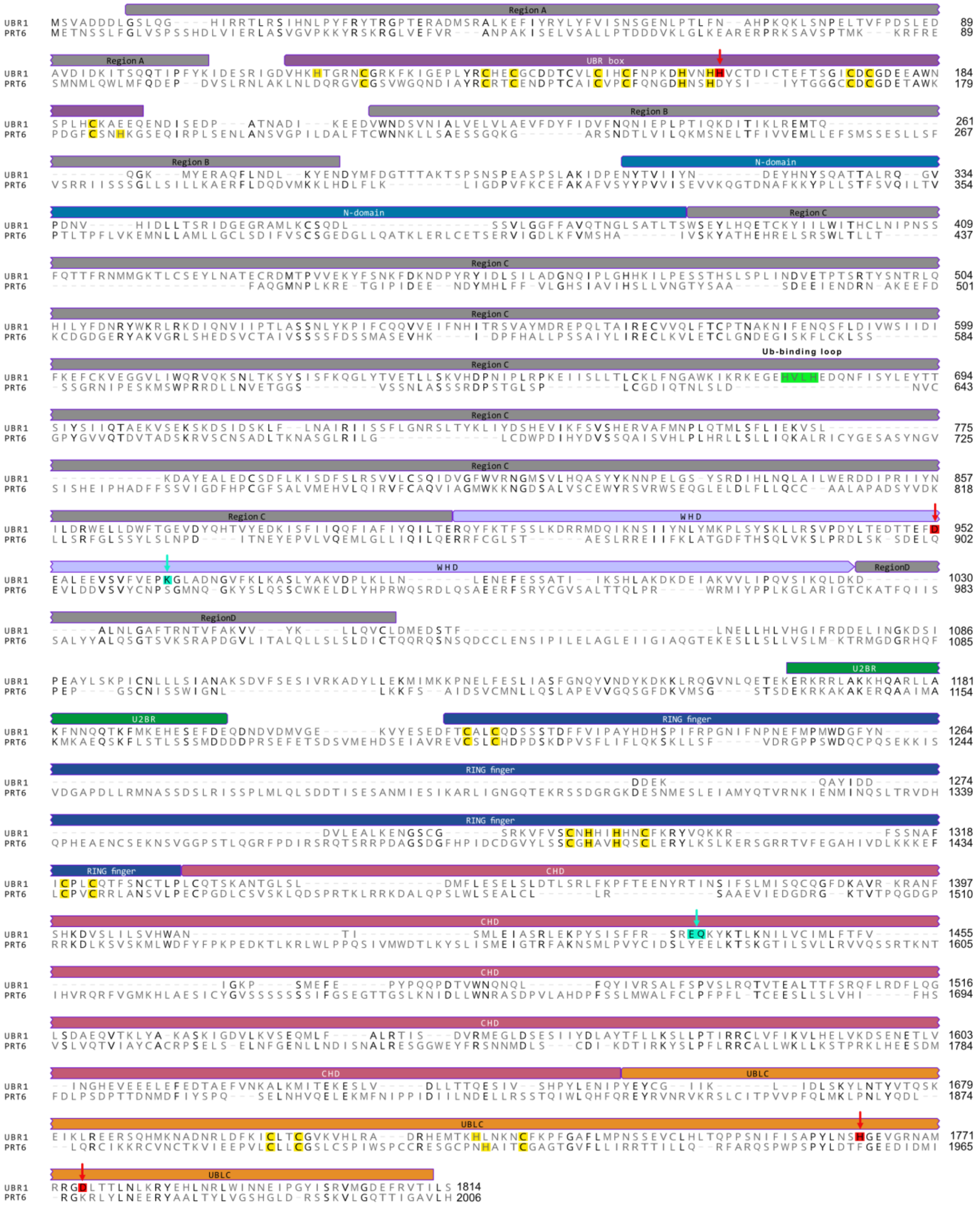

Supplementary Figure S2A. Amino acid sequence alignment of Saccharomyces UBR1 (UniProt accession number P19812) and Arabidopsis PRT6 (UniProt accession number F4KCC2). Saccharomyces UBR1 domains are annotated according to the cryo-EM structure (PDB 7MEX) [51]. The zinc coordinating residues of the UBR-Box, RING finger, and UBLC domains are indicated in yellow. Red arrows indicate a putative zinc coordination site involving UBR Box (His161), WHD (Asp952) and the UBLC domain of UBR1 (His1763, Asp1775). A green box indicates a Ub binding loop in UBR1. Cyan arrows indicate UBR1 residues involved in Ub chain elongation.

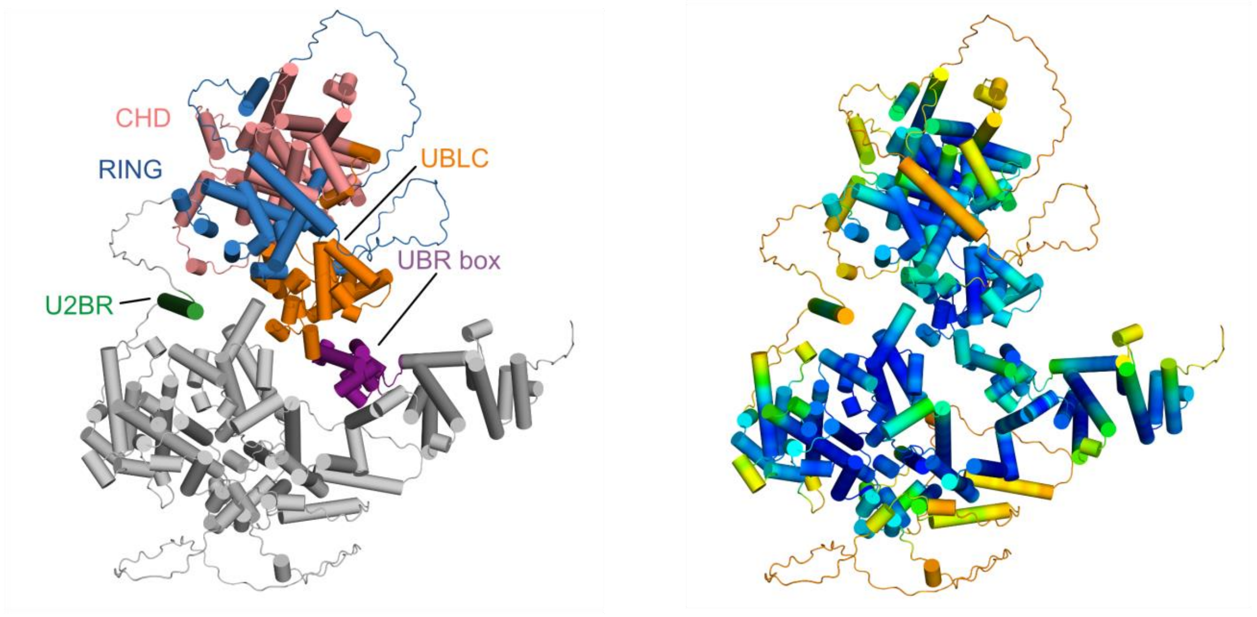

Supplementary Figure S2B. AlphaFold2 model of Arabidopsis PRT6 (AlphaFold Protein Structure Database: AF_AFF4KCC2F1). Left: The predicted domain architecture of PRT6. Right: model coloured by predicted local distance difference test (pLDDT) score. Colours from high to low pLDDT; blue, cyan, green, yellow, orange.

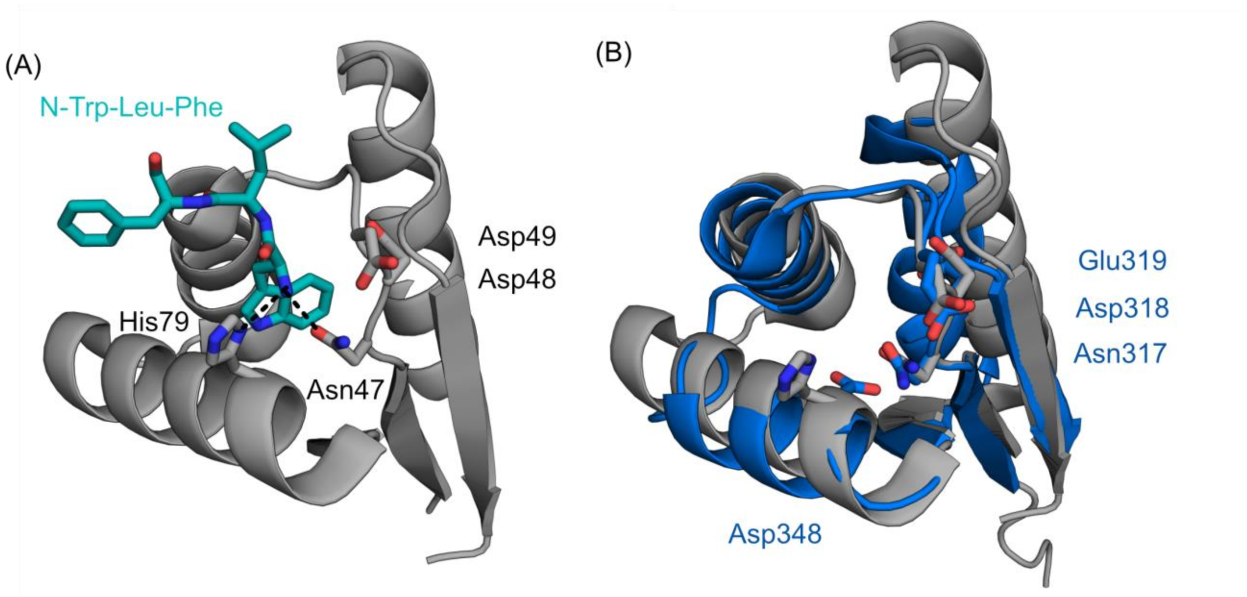

C

```
PRT6 YYPVVISEVVKQGTDNAFKKYPLLSTFSVQILTVPTLTPFLVKEMNLLAMLLGCLSDIFVSCSGEDGLLQATKLERLCETSERVIGDLKFVMSHAI 448
UBR1 NYTVIIYN-------DEYHNYSQATTALRQG--VPDN----VHI-DLLTSRIDGEGRAMLKCSQDLS---------------SVLGGFFAVQTNGL 382
3O1F MYKVILVN-------DDYTPMEFVIDVLQKF--FS-YDVERATQ-LMLAVHYQGKAICGV-FTAEVA-------ETKV----AMVNKYARENEHPL  99
3GQ1 LYRVLILN-------DDYTPMEFVVYVLERF--FN-KSREDATR-IMLHVHQNGVGVCGV-YTYEVA-------ETKV----AQVIDSARRHQHPL 112
4O2X AWKVILYN-------DDIHNFTYVTDVIVKV--VGQISKAKAHT-ITVEAHSTGQALILS-TWKSKA-------EKYC----QELQ------QNGL 176
7D34 PYRVILHN-------DNFNKREYVVQVLMKV--IPGMTVDNAVN-IMQEAHINGLAVVIV-CAQADA-------EQHC----MQLR------GNGL 148
```

Supplementary Figure S3. The UBR1 N-domain is structurally similar to ClpS proteins. (**A**) The *Caulobacter crescentus* ClpS protein bound to an N-degron (PDB 3GQ1). The α-amino group of the substrate interacts directly with His79 and Asn47. Hydrogen bonds are indicated with dashed lines. (**B**) Superposition of the N-domain of Saccharomyces UBR1 (blue, PDB 7MEX) and *C. crescentus* ClpS (grey, PDB 3GQ1). (**C**) Amino acid sequence alignment of PRT6 with representative ClpS domains with known three-dimensional structures. Sequences are Arabidopsis PRT6 (UniProt accession number F4KCC2), Saccharomyces UBR1 (PDB 7MEX), *Escherichia coli* ClpS (PDB 3O1F), *C. crescentus* ClpS (PDB 3GQ1), *Plasmodium falciparum* ClpS (PDB 4O2X), and Arabidopsis ClpS1 (PDB 7D34). Conserved binding pocket residues are indicated in red.

(A) UBR-Box

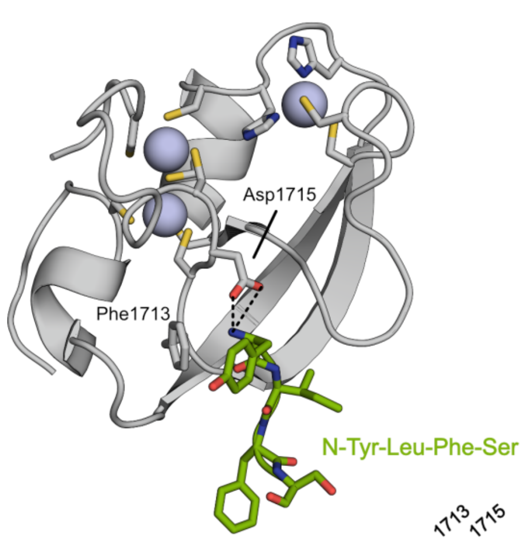


```
                                                                  1713 1715
                                                                     |  |
UBR4  KLCTFTITQKEFMNQHWYHCHTCKMVDGVGVCTVCAKVCHKDHEISYAKYGSFFCDCGAKED--GSCLALVK   1729
BIG   KVCTFTSSGSNFMEQHWYFCYTCDLTVSKGCCSVCAKVCHRGHRVVYSRSSRFFCDCGAGGVRGSSCQCLKP   1364
```

(B) DOC-1

```
UBR4   DFFEHNQQLTDVEFGGNDLLQVYNAQQIKHRLNSTGMYVANTKPGGFTIEISNNNSTMVM   2349
BIG    DFFERAFCITADVRLGSDAIRNGDSEGAKQSLASEDGFIESPSPVGFKISVSNPNPDIVM   2214
       ****     *     * *          *  * *         * ** *  ** *   **

UBR4   TGMRIQIGTQAIERAPSYIEIFGRTMQLNLSRSRWFDFPFTREEALQADKKLNLFIGASV   2409
BIG    VGIRMHVGTTSASSIPSEVTIFQRSIKMDEGMRCWYDIPFTVAESLLADEDVVISVGPTT   2274
        * *   **      **   ** *          * * ***  * * **       *

UBR4   DPAGVTMIDAVKIYGKTKEQFGWPDEPPEEFPSASVSNICPSNLNQSNGTGDSDSAAPTT   2469
BIG    SGTALPRIDSLEVYGRAKDEFGWKEKMDAVLDMEARV--LGHGLLL-P--GSSK-KRALA   2328
              **    ** *   ***                    *      * *

UBR4   TSGTVLERLVVSSLEALESCFAVGPIIEK-       2498
BIG    QSASMEEQVIADGLKLLSIYYSVCRPRQEV       2358
        *    *      *  *     *
```

(C) ZZ domain

```
BIG    CDGCSTVPILRRRWHCTVCP---DFDLCEACYEVLDA--DRLPPPHTRDHPM 2643
p62    CDGC-NGPVVGTRYKCSVCP---DYDLCSVCEGKGL---------HRG-HTK 165
PRT1   CDSCGVYPIIGDRYRCKDCKEEIGYDLCKDCYETPSKVPGRFNQQHTPDHRL 362
KCMF1  CDACLKGNFRGRRYKCLICY---DYDLCASCYESGATTT-----RHTTDHPM 52

UBR4   MVSESMVLETAENVNNGNPSPLEALLAGAEGFPPMLDIPPDADDETM 2815
```

(D) Cysteine Rich (CR) region

```
UBR4   CPRCSASVPANPGVCGNCGENVYQCHKCRSINYDEKDPFLCNAC 3703
BIG    CPRCSRPVTDKHGICSNCHENAYQCRQCRNINYENLDSFLCNEC 3486
```

(E) Calmodulin binding region

```
UBR4  GKAPSKSELRHLYLTEKYVWRWKQFLSRRG 4113
BIG   GASQKSRTHRSDFLALKYTLRWKRRSSRTS 3904
      *        *   *  **  ***   **
```

Supplementary Figure 4. Comparison of human UBR4 (UniProt accession number Q5T4S7) and Arabidopsis BIG (UniProt accession number Q9SRU2). BIG residues are numbered according to [103]. (**A**) The UBR-Box of human UBR4 (PDB 8J9R, grey cartoon) in complex with an N-degron (green sticks). Zinc coordinating residues and two residues which are essential

for substrate binding are shown as sticks. Asp1715 hydrogen bonds with the alpha-amino group of the substrate and Phe1713 is essential for both type I and II degron binding. An amino acid sequence alignment of UBR4 and BIG UBR-Box domain highlighting zinc coordinating residues (red). (**B**) An amino acid sequence alignment of the DOC-1 region of UBR4 and BIG, asterisks indicate identical residues. (**C**) An amino acid sequence alignment of the ZZ-domains from Arabidopsis BIG, human p62/sequestosome 1 (UniProt accession number Q13501), Arabidopsis PRT1(UniProt accession number Q8LBL5) and human KCMF1 (UniProt accession number Q9P0J7). The putative zinc coordinating residues are in red. The equivalent region in human UBR4 lacks cysteines. (**D**) An amino acid sequence alignment of the UBR4 and BIG cysteine rich (CR) region, conserved cysteines are in red. (**E**) An amino acid sequence alignment of the UBR4 and BIG calmodulin binding region, asterisks indicate identical residues.
